# Liposomal Tumor Targeting in Drug Delivery Utilizing MMP-2- and MMP-9-Binding Ligands

**DOI:** 10.1155/2011/160515

**Published:** 2010-12-29

**Authors:** Oula Penate Medina, Merja Haikola, Marja Tahtinen, Ilkka Simpura, Sami Kaukinen, Heli Valtanen, Ying Zhu, Sari Kuosmanen, Wei Cao, Justus Reunanen, Tuula Nurminen, Per E. J. Saris, Peter Smith-Jones, Michelle Bradbury, Steven Larson, Kalevi Kairemo

**Affiliations:** ^1^Department of Radiology, Sloan Kettering Institute for Cancer Research, 1275 York Ave., New York, NY 10065, USA; ^2^Division of Pharmaceutical Technology, University of Helsinki, P.O. Box 56, FI-00014, Finland; ^3^CTT Cancer Targeting Technologies Ltd., Viikinkaari 4 C, FI-00790 Helsinki, Finland; ^4^Department of Food and Environmental Sciences, University of Helsinki, P.O. Box 56, FI-00014, Finland; ^5^International Comprehensive Cancer Center Docrates, Saukonpaadenranta 2, FI-00180 Helsinki, Finland

## Abstract

Nanotechnology offers an alternative to conventional treatment options by enabling different drug delivery and controlled-release delivery strategies. Liposomes being especially biodegradable and in most cases essentially nontoxic offer a versatile platform for several different delivery approaches that can potentially enhance the delivery and targeting of therapies to tumors. Liposomes penetrate tumors spontaneously as a result of fenestrated blood vessels within tumors, leading to known enhanced permeability and subsequent drug retention effects. In addition, liposomes can be used to carry radioactive moieties, such as radiotracers, which can be bound at multiple locations within liposomes, making them attractive carriers for molecular imaging applications. Phage display is a technique that can deliver various high-affinity and selectivity peptides to different targets. In this study, gelatinase-binding peptides, found by phage display, were attached to liposomes by covalent peptide-PEG-PE anchor creating a targeted drug delivery vehicle. Gelatinases as extracellular targets for tumor targeting offer a viable alternative for tumor targeting. Our findings show that targeted drug delivery is more efficient than non-targeted drug delivery.

## 1. Introduction

Liposomal nanotechnology provides a versatile platform for exploring several approaches that can potentially enhance the delivery and targeting of therapies to tumors. As a biodegradable and essentially nontoxic platform, liposomes can be used to encapsulate both hydrophilic and hydrophobic materials and be utilized as drug carriers in drug delivery systems (DDSs). In addition, liposomes can be used to carry radioactive moieties, such as radiotracers, which can be bound at multiple locations within liposomes, making them attractive carriers for molecular imaging applications. In this study, gelatinase-binding peptides were attached to liposomes for synthesizing a targeted drug delivery vehicle.

For active targeting or drug delivery applications or both, intraliposomal encapsylation of multiple targeting agents or therapies can be (i) to the lipid bilayer, which can bind hydrophobic conjugates; (ii) to hydrated compartments for water-soluble components; (iii) by covalent binding directly or by utilizing spacer to the outer lipid leaflet [1]. Delivery of these nanoformulations to the reticuloendothelial system (RES) is readily achieved since, given their larger size, the RES traps most conventional liposomes that are not shielded by polyethylene glycol chains (PEGs) or other similar steric water carrying substance. RES uptake can be increased by altering particle surface chemistry and charge, for instance, by adding positively charged lipids or biologically activating proteins or sugars on the surface of the liposomes. For purposes of agent delivery to target organs other than the RES, long-circulating liposomes have been developed by modifying the liposomal surface [2]. Determination of the *in vivo* biodistribution and targeting kinetics of liposome-encapsulated drugs is required for the assessment of drug bioavailability. 

The most commonly used nanoformulated drug is Caelyx/Doxil, a liposomal doxorubicin product. It has nearly supplanted doxorubicin in the therapy of ovarian cancer, breast cancer, and Kaposi's sarcoma. It differs from the former generation liposomal delivery systems, as the outer surface of Caelyx/Doxil is coated with PEG chains that protect the liposomes from being opsonized by components of the immune system in the circulation. These stealth-type liposomes have longer circulation half-times than those for uncoated liposomes. In addition, they are safer than the native drugs themselves (e.g., Caelyx/Doxil is not cardiotoxic, a major concern for native doxorubicin delivery).

For cancer-based applications, peptides that can selectively detect and target metastatic disease and tumor invasive potential may offer critical prognostic information. Metastatic invasion is promoted by the attachment of tumor cells to the extracellular matrix, the degradation of matrix components by tumor-associated proteases, and the cellular movement into the area modified by protease activity. Matrix metalloproteases (MMPs) represent a family of enzymes capable of degrading the basement membrane and extracellular matrix (ECM), thus contributing to tissue remodeling and cell migration [3–5]. This family of enzymes can cleave ECM proteins, as well as alter the integrity of basement membranes that serve as barriers to cell movement. This is normally a tightly regulated process, with the presence of activated metalloproteinases occurring only under specific conditions. 

MMPs may be divided into subgroups, one comprised of type IV collagenases (gelatinases) such as MMP-2 and MMP-9, which play major roles in tumor growth, angiogenesis, and metastatic disease. These gelatinases degrade type IV collagen (and its breakdown product, gelatin) and comprise the primary structural component of the ECM, enabling tumor cells to gain access to the rest of the body. Overexpression and/or prognostic significance of gelatinases have been examined in a range of cancer types, including ovarian cancer [6–9], endometrial and cervical cancer [10, 11], and breast cancer [12, 13]. High expression levels of gelatinases in breast and ovarian cancers, for instance, are known to be associated with unfavorable prognoses. 

In this study, binding peptides (BPs) extracted from MMP-9 were attached to liposomes for synthesizing a targeted drug delivery vehicle. Downregulation of MMP-9 is known to exert inhibitory effects on endothelial cell migration and tube formation [14]. Intriguingly MMP-2 has been shown to dock on tumor cell-surface integrins, which makes gelatinases even more interesting as a target [15]. Adenoviral-mediated MMP-9 downregulation was shown to retard endothelial cell migration in cell wounding and spheroid migration assays, resulting in reduced capillary-like tube formation [16]. MMP-2- or MMP-9-deficient mice were found to exhibit abnormal angiogenic properties [17]. Further, in human gliomas, immunohistochemical findings suggested that neoplastic and endothelial cells expressing MMP-9 protein may be associated with tumoral angiogenesis [18]. 

**Figure 1:**
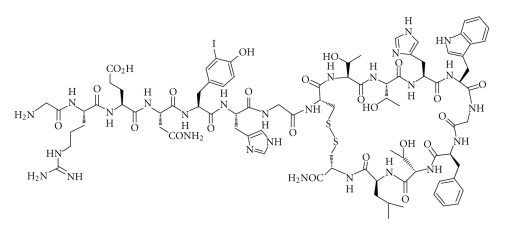
Chemical structure of ^125^I-CTT2-peptide. CTT2-peptide is a 17-amino acid peptide with a disulphide bridge between the two cysteines. The amino terminal end of the peptide is amidated to increase its stability. Upon iodination, peptide labeling occurs on the aromatic ring of the tyrosine amino acid.

One of the first known specific gelatinase inhibitors, a cyclic MMP-9-binding peptide identified by random phage display libraries (i.e., CTTHWGFTLC peptide later CTT1), has previously been shown to have high affinity not only to MMP-9, but also to MMP-2 [19]. The peptide actively inhibits endothelial and tumor cell migration *in vitro* and tumor progression in *in vivo* murine models [19]. Specifically, CTT-displaying phages block the formation of new blood vessels, resulting in tumor size reductions and prolonged overall survival. These findings highlight the potential of CTT peptide for targeting chemotherapeutics or other imaging probes to the tumor neovasculature. CTT-peptide has been used for liposomal drug delivery *in vitro *and *in vivo* [20, 21] and has been shown to be effective in improving selective localization of chemotherapies such as doxorubicin in human tumor cells. In this work we modified a CTT peptide with a peptide linker that bears a tyrosine moiety for iodination procedures and attached several additional amino acids (GRENYHG) to enhance freedom of peptide binding in order to create GRENYHGCTTHWGFTLC-peptide (i.e., CTT2-peptide) (Figure 1). The synthesis of CTT2-peptide enabled us to retain bioactivity that would otherwise not be present if CTT peptide itself was directly linked to lipids or PEG spacers. This is the first study, to our knowledge, that has utilized a peptide derived from a synthetic phage display library for constructing a more selective liposomal delivery system for targeting extracellular target molecules. 

We initially present the synthesis of PEG-PE-CTT2 peptide-bound micelles and liposomes. The feasibility of utilizing micellar and liposomal nanoformulations as therapeutic delivery vehicles to achieve efficacy in ovarian carcinoma models was explored by attaching the radioiodinated CTT peptide tracer, ^125^I-CTT2 peptide, to these platforms and loading them with doxorubicin, an inherently fluorescent chemotherapeutic agent. Biodistribution studies of both targeted nanoformulations were performed in normal and immunosuppressed subcutaneous human xenograft models using the CTT2-peptide. 

## 2. Materials and Methods

**Reagents**

All reagents, unless stated otherwise, were obtained from Sigma-Aldrich (St Louis, Missouri, USA) and culture media from Gibco Life Technologies (Paisley, Scotland). PEG-PE-NHS was from Avanti Polar Lipids Inc. (Alabama, USA) as all the other lipids used in this study.

### 2.1. Synthesis of Peptides

Peptides were synthesized on an Applied Biosystems 433A (Foster City, CA, USA) automatic synthesizer using Fmoc-chemistry. For disulfide generation, peptides were dissolved at 1 mg/ml in 0.05 M ammonium acetate (pH 8) and mixed with H_2_O_2_ for 40 min at room temperature so that 0.5 ml of 3% H_2_O_2_ was added per 100 mg peptide. The peptides were purified by reversed phase HPLC, and the molecular weight was identified by mass spectrometry analysis. 

### 2.2. Synthesis of DSPE-PEG3400-CTT2

**Figure 2:**
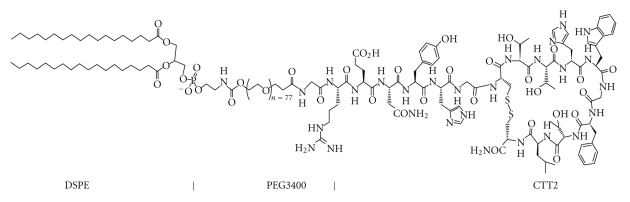
Chemical structure of CTT2-PEG3400-DSPE. CTT2-PEG3400-DSPE was synthesized by coupling CTT2-peptide to PEG3400-DSPE, followed by purification of the reaction product from the initial mixture.

Coupling bioactive peptides to PEGylated lipids can alter the pharmacokinetics and dynamics of these peptides. For pharmaceutical formulation purposes, CTT2-peptide (Figure 1) was covalently attached to the PEG phospholipid (Figure 2).

In this procedure, the peptide called CTT2 (cyclo-GRENYHGCTTHWGFTLC-NH_2_) was covalently attached to PEG phospholipids through the chemical reaction between the terminal amine of the peptide and the functional NHS (hydroxysuccinimidyl) group at the end of the PEG phospholipid polymer chain. The reaction between the terminal amine and the active succinimidyl ester of the PEG carboxylic acid produced a stable amide linkage. Different molar ratios of the peptide and the PEG phospholipid, as well as the reaction times, were varied to optimize the coupling reaction. Up to several hundred CTT2-PEG-lipid molecules can be attached to the surface of each liposome.

CTT2 peptide (8.8 mg) and DSPE-PEG3400-NHS (100 mg) were dissolved in 2 milliliters (ml) dimethylformamide. CTT2 peptide solution (500 *μ*l) was mixed with 600 *μ*l of DSPE-PEG3400-NHS solution and incubated for 21 hours (hrs). Samples were then precipitated by addition of diethylether and centrifuged (13200 rpm for 10 min). The supernatant was decanted and the solid residue was stored at −70°C. 

For all studies, samples were reconstituted by adding 100 *μ*l methanol and 25 *μ*l of 1 M sodium hydroxide, followed by 250 *μ*l of 1% TFA in water after two hours. Analysis of these samples was performed after centrifugation (4200 rpm 20 min) using a C-18 RP-HPLC by initially precipitating the purified product with excess diethylether. The solid residues were dissolved in 1500 *μ*l methanol and analyzed by thin layer chromatography (TLC). Reaction yields for CTT2 peptide- DSPE-PEG3400-NHS coupling averaged 6.0 mg.

### 2.3. Preparation of Liposomes

#### 2.3.1. CTT2-Micelles

Monomers or CTT2-PEG3400-DSPE (i.e., CTT2-PEG-lipid) spontaneously formed micelles *∼*14 nm in diameter (i.e., CTT2-micelles) in aqueous solution, with DSPE lipid chains forming the hydrophobic core and PEGylated CTT2-peptide forming the hydrophilic surface of the micelle. CTT2-micelles were covalently labeled with radioiodine, I-125 (^125^I, half-life = 13 hrs), to determine time-varying tissue distributions and tumor uptakes. Radiochemical purity of *∼*90% was achieved. 

#### 2.3.2. CTT2-SL Liposomes

CTT-2-peptide-targeted liposomes were synthesized either by incorporating CTT2-PEG-lipid onto the surface of commercially available liposomes or by combining CTT2-micelles with liposomal formulations. Prior studies have shown that incubation of certain lipids with liposomes can result in intraliposomal inclusion of these lipids as a consequence of micellar-liposomal fusion [23, 24]. This spontaneous process, occurring when lipid concentrations exceed critical micellar concentrations (CMC), has been used as a postinsertion technique with preformed liposomes to produce immunoliposomes [25] and liposomes coated with peptides or oligosaccharides [26, 27]. CTT2-micelles were combined with the commercially available nanoformulated drug, Caelyx (PEGylated liposomal doxorubicin HCl), to form CTT2-peptide-targeted Caelyx (CTT2-SL liposome). This method provides a CTT2-PEG-lipid content of *∼*0.2% of all lipids on the resulting liposome surface; CTT2-peptide-lipid concentrations are essentially the maximum achievable concentrations using CTT2-micelle methodologies as Caelyx liposomes are PEGylated. The lipid components of CTT2-SL liposome and Caelyx that were used for these studies are listed in Table 2.

CTT2-SL liposomes were made by pipetting the above-mentioned lipid mixture except the CTT2-PEG-lipid, to a round bottomed flask, dried under nitrogen and lyophilized for 2 h to remove trace amounts of chloroform. Doxorubicin liposomes were prepared by using standard pH gradient technique [1]. 

To synthesize CTT2-PEG-3400-DSPE Caelyx/doxil-liposomes, CTT2-PEG-DSPE (1 mg) was suspended in 400 *μ*l of buffer (100 mM histidine, 55 mM sucrose, pH 6.5), and 100 *μ*l of this CTT2-PEG-DSPE micelle suspension was added to 1 ml Doxil/Caelyx solution or internally prepared similar to doxil-liposomes (Ortho Biotech). *In vivo* murine studies were performed after incubating the mixture for 30 min at 60°C. 

The incorporation efficiency, the percentage of total activity contained in the liposome fractions, was measured by using radioisotope-labeled peptide and gel filtration to separate the unreacted micelle from the liposome; optimal reaction conditions were found to be 60°C at 30 min (nearly 100% efficient). 

The doxorubicin leakage from the liposomes after the incorporation experiments was determined by comparing the amount of free doxorubicin versus liposome-bound doxorubicin before and after the experiment. The leakage was found to be minimal (the leakage before the incorporation was in average 4.5% and after the reaction in average 4.2%).

### 2.4. Radiolabeling of Peptides

Radiolabeling of peptides and all liposomal formulations with iodine-125 (^125^I) was performed using the IODOGEN (Pierce, Rockford, IL). The CTT2-PEG3400-DSPE peptide was labeled with ^125^I using iodogen as a catalyst. 5 MBq of Na125I (Amersham, Buckinghamshire, England) in 0.5 ml PBS was added to a tube containing 10 *μ*g dried iodogen and 100 *μ*g CTT2-PEG3400-DSPE peptide construct. The mixture was incubated for 20 min at room temperature. The ^125^I-bound particle fractions were purified by elution from PD-10 columns. The activity of the peptide was determined in a gamma counter (Cobra II, Packard Instruments).

### 2.5. Animal Models and Tumor Inoculation

The mice were cared for according to the instructions of the animal facility, and the experiments were approved by an ethical committee of Helsinki University, Finland. Male athymic nu/nu mice (6–8 weeks old, Harland) were provided with water and maintained on regular diets ad libitum. Subcutaneous human serous ovarian carcinoma (OV-90) xenograft models were generated by coinjecting equal volumes of cells (*∼*5×10^6^/100 *μ*l phosphate buffered saline, PBS) and matrigel subcutaneously into the hindlegs of nude mice. Average tumor volumes of 65 mm^3^–200 mm^3^  were used for all studies.

### 2.6. In Vivo Biodistribution and Pharmacokinetics

Following single i.v. tracer doses of purified ^125^I-CTT1-peptide (*∼*40 *μ*g/mouse),  ^125^I-CTT2-peptide (*∼*40 *μ*g/mouse), or CTT2-micelles (200 *μ*g/mouse; 200 kBq), the percentage of the injected dose per gram of tissue (%ID/g) values, corrected for radioactive decay to the time of injection, were measured in tumor and major tissues/organs (heart, liver, kidney, lung, muscle, brain, spleen, and tumor) by sacrificing groups of normal mice or mice bearing serous ovarian hindleg xenografts (OV-90) at specified time points. 

Additional distribution data was measured in immunosuppressed mice (*n* = 6/group) bearing subcutaneous human mucinous ovarian tumors (A2780) using single bolus injections of CTT2-SL liposome or Caelyx (9 mg/kg, calculated doxorubicin equivalents). Lyophilized tissue and plasma were extracted in acid alcohol, and their doxorubicin concentrations were determined using a Varian spectrofluorometer. Doxorubicin fluorescence intensities (a.u.) were measured at 590 nm using excitation wavelengths of 470 nm, and comparing these intensities against standard samples containing known amounts of doxorubicin. Doxorubicin concentrations in tumor (*μ*g doxorubicin per gram dry tissue) were expressed at each time point when delivered as CTT2-SL liposome or Caelyx. 

### 2.7. Efficacy Studies

#### 2.7.1. Doxorubicin Administered as CTT2-SL Liposomes and Caelyx

Therapeutic efficacy studies were conducted in subcutaneous A2780 xenografts using doxorubicin, administered as either CTT2-SL liposomes or Caelyx. Commercially available nonliposomal (“free”) drug (i.e., doxorubicin) and saline dilution buffer were used as treatment controls. A2780 ovarian cancer cells (5×10^6^ in 100 *μ*l PBS) were injected subcutaneously into the posterior flanks of NMRI nude mice (*n* = 40). Mice received i.v. bolus injections of CTT2-SL liposome, Caelyx, doxorubicin, and buffer. CTT2-SL liposomes were injected when tumor volumes reached 65 mm^3^, while administration of Caelyx and doxorubicin to different xenograft mice was offset in time from CTT2-SL liposomes by 3 and 6 days, respectively. Doxorubicin, CTT2-SL liposomes, and Caelyx were injected at doses of 9 mg/kg each. Mouse body weights were monitored throughout the study period.

Aforementioned treatments were used to collect two independent biodistribution data sets in immunosuppressed OV-90 xenograft mice (*n* = 5/group). In one set of studies, CTT2-SL liposomes were injected using lower doses of doxorubicin (5 mg/kg) compared to Caelyx (9 mg/kg). Doxorubicin was also administered to a second group of mice (n-3 per group) in the form of CTT2-SL-DSPE-PEG3400 liposomes or CTT2-Caelyx-like liposomes. These latter formulations were bolus injected using 9 mg/kg (calculated doxorubicin equivalents). Harvesting, weighing, and counting of blood, tumor, and major organs in a scintillation *γ*-counter were performed for all studies at specified time points. Doxorubicin was extracted from these formulations, and concentrations were analyzed using HPLC.

## 3. Results and Discussion

### 3.1. Biodistribution and Clearance Studies

The initial reason to create the CTT-2 peptide was to make a peptide that was more easily iodinated and that offered a spacer that was comfortably used for linking purposes without destroying the bioactivity of the peptide. 

**Figure 4:**
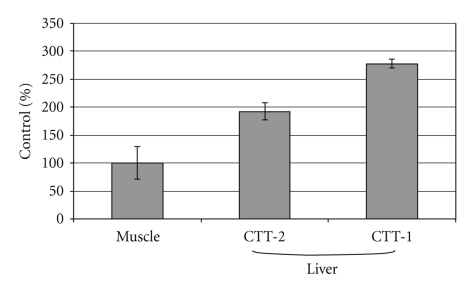
Hepatic accumulation of ^125^I-CTT2-peptides in normal mice. Liver accumulation of peptides per gram of tissue in normal mice (*n* = 5) relative to muscle (control). All values are expressed as the percentage of the control (% control) ± SD.

In nontumor-bearing mice, greater liver accumulation of the CTT1-peptide was observed than with the CTT2-peptide (Figure 4). This was thought to be secondary to the increased relative hydrophobicity of the former peptide construct. All other tissues analyzed demonstrated no significant difference in the magnitude of uptake of these peptides (data not shown) [21]. The CTT2-peptide was thus selected for further studies given its more rapid hepatic clearance. 

**Figure 5:**
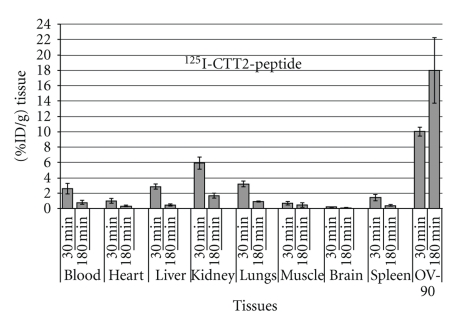
Tissue distribution of a single dose of ^125^I-CTT2-peptide in immunosuppressed OV-90 xenograft mice. Blood and major organs/tissues were collected at 0.5 hr and 3 hrs p.i. ^125^I-CTT2-peptide (40 *μ*g/mouse, *n* = 5) and their radioactivities were measured. Results are expressed as percentage of injected dose per gram tissue (%ID/g). All values are given as mean ± SD.

In OV-90 xenograft models, substantially higher uptake of ^125^I-CTT2-peptide was measured in all organs/tissues (Figure 5), particularly in the xenograft, with tumor-to-blood ratios *∼*23 detected at 3 hrs postinjection (p.i.). This coupled with the poor prognosis of this disease in humans, showed the potential to improve treatment response using CTT2-peptide targeted delivery, and the need to ensure controlled and sustained drug release led us to extend this model to investigate tumor uptake with micellar and liposomal formulations (CTT2-micelles and CTT2-liposomes). The amount of CTT2-bound peptide available for liposomal targeting activity was found to be 500 based on the measured average size and surface area of the resulting peptide-bound liposomal product by dynamic light scattering and the aforementioned reaction conditions. 

For doxorubicin-containing liposomes, doxorubicin leakage after peptide attachment was assessed by comparing free and liposomal doxorubicin on the basis of fluorometric analysis. Leakage was found to be minimal, with leakage before and after incorporation averaging 4.5% and 4.2%, respectively. 

**Figure 6:**
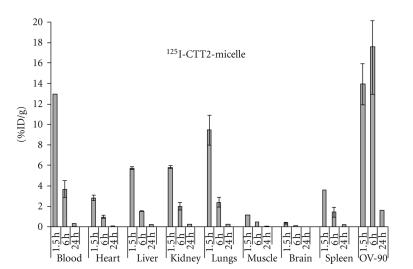
Tissue distribution of ^125^I-CTT2-micelle in OV-90 tumor mice. %ID/g values after i.v. injection of CTT2-micelles (200 *μ*g/mouse, *n* = 5). All values are expressed as mean ± SD.

 Both OV-90 and mucinous ovarian carcinomas (A2780) were thus selected as xenograft models for subsequent nanoformulation studies. In OV-90 tumor mice, clear targeting of CTT2-micelles was observed, reaching maximum values of 17.6% of the injected dose per gram (%ID/g) of tumor at 6 hrs p.i. (Figure 6).

**Figure 7:**
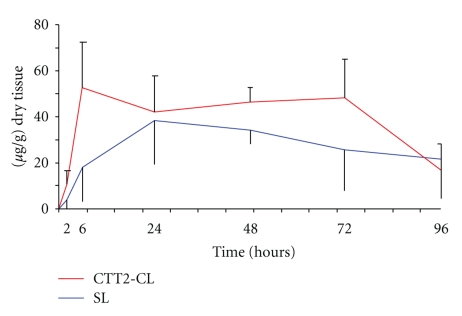
Comparison of doxorubicin concentrations in tumors after a single i.v. injection of CTT2-SL liposome or Caelyx. A2780 xenografts (*n* = 6) were collected at 2, 6, 24, 48, 72, and 96 hours after CTT2-liposome or Caelyx injection, and their doxorubicin content was measured using spectrophotometry. Results are shown as *μ*g drug per gram of dry tissue. All values are expressed as the mean ± SD. The differences in each time point are near significant. The overall difference in the AUC is significant.

Doxorubicin concentrations (*μ*g doxorubicin per gram dry tissue), in the form of CTT2-SL (targeted) and SL (nontargeted Caelyx/Doxil) liposomes, were measured as a function of time p.i. in A2780 xenografts, as shown in Figure 7. Doxorubicin was delivered more efficiently and at a faster rate to tumors using CTT2-SL liposomal formulations compared to Caelyx, with significantly elevated tumoral levels of doxorubicin observed 3 days after drug injection.

**Figure 8:**
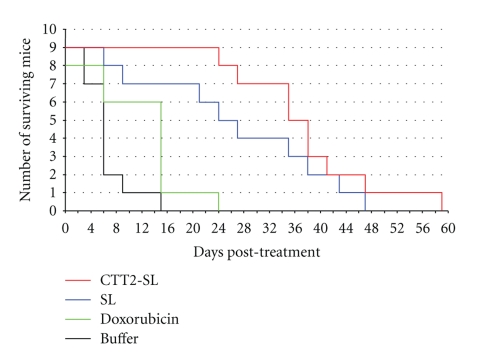
Kaplan-Meier plot of the survival of tumor bearing mice. Mice were treated with doxorubicin (9 mg/kg), administered either as CTT2-SL liposome or Caelyx. Controls were injected with doxorubicin (9 mg/kg) or saline dilution buffer. Injections for each treatment group were made at day 0, day 3 and day 6, respectively.

CTT2-SL liposomal antitumor efficacy data following i.v. bolus injections of CTT2-SL liposome, Caelyx, doxorubicin, and buffer in A2780 xenografts is shown in Figure 8. Live mice exhibiting tumor sizes exceeding 1000 mm^3^ were sacrificed, including those at days 15 and 24 following i.v. administration of buffer or doxorubicin, respectively. Importantly, 80% of mice treated with CTT-SL liposomes and 50% treated with Caelyx were alive at 24 days following initiation of treatment. Treatment with CTT2-SL liposomes was therefore found to increase mean survival times of mice by 38% from 27.9 to 38.6 days. 

**Figure 9:**
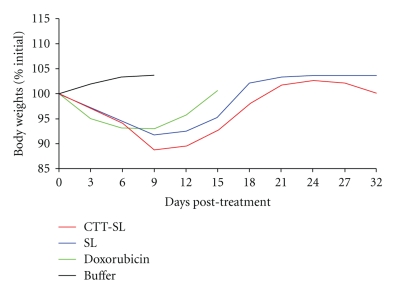
Mouse body weight changes in each treatment group during the first 32 days of the trial. Mice were treated with 9 mg/kg doxorubicin (calculated doxorubicin equivalents) or saline dilution buffer at day 0, 3 and 6. All values are expressed as mean of 9 mice.

Mouse body weights were monitored throughout the study period (Figure 9). Each doxorubicin regimen (CTT2-SL liposome, Caelyx, and Doxorubicin) induced a slight weight decrease with a maximum loss of about 10% at day 9. However, one week later, body weights returned to initial levels.

**Figure 10:**
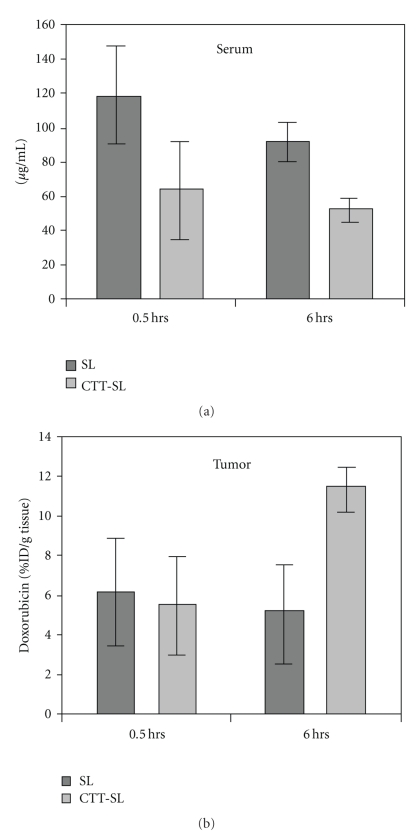
Concentrations of doxorubicin in (a) serum and (b) OV-90 xenografts in mice treated with CTT2-SL liposome and Caelyx at 0.5 and 6 hours. Data are represented as a mean of 5 mice ± SD.

Given the overall improved survival found following treatment of A2780 xenografts with CTT2-SL liposomes, studies were extended to assess treatment response in OV-90 xenograft models. As seen in Figure 10, despite the lower doses of CTT2-SL liposomes administered, efficient targeting, along with rising concentrations of doxorubicin was measured using this serous ovarian model (Figure 10(b)) over a 6-hr time interval relative to the untargeted formulation. 

**Figure 11:**
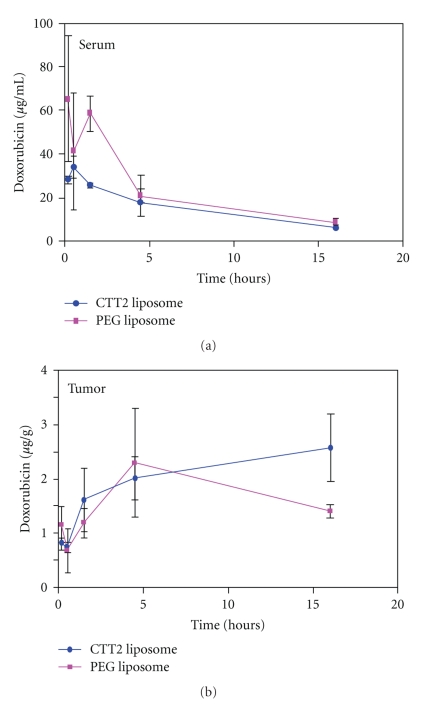
Serum doxorubicin levels. Concentration of doxorubicin in (a) serum and (b) OV-90 xenograft mice (*n* = 3) treated with CTT2-SL-DSPE-PEG3400. Data are represented as a mean ± SEM.

Additional serum and tumor uptake measurements conducted with CTT2-SL DSPE-PEG3400 liposomes are shown in Figure 11. Initial serum doxorubicin concentrations were found to be lower for CTT2-SL-DSPE-PEG3400 liposomes than for untargeted liposomes (i.e., PEG-liposomes), but the overall kinetic profile of the two liposomal formulations was essentially equivalent over time.  Figure 11(b)demonstrates time-dependent changes in the total amount of doxorubicin found in tumor tissue. Unlike prior studies performed with CTT2-SL liposomes and Caelyx, where maximal differences in tumor tissue accumulation were detectable at 6 hours, doxorubicin accumulations in the present study were similar for both liposomal products at earlier time points. However, at 16 hours p.i., a clear difference is observed in the accumulated doxorubicin tumor concentrations, confirming earlier findings that efficacy improves with CTT2-peptide-bound liposomal delivery systems. The extended times of accumulation may be a consequence of the different liposomal formulations used. Doxorubicin concentrations, in the form of CTT2-SL-DSPE-PEG3400 liposomes, continued to rise at later time points, as against the notable decreases in tumor concentrations observed with the untargeted CTT2-Caelyx-like liposomes. Future kinetic studies should monitor time-varying changes in tumor doxorubicin concentrations (in the form of CTT2-peptide targeted liposomes) at delayed time intervals (i.e., >16 hrs p.i.) in order to determine whether antitumor efficacy studies could benefit from employing a dosing regimen reflecting longer, sustained tumor concentrations.

## 4. Conclusions

Gelatinases, as extracellular targets, offer a viable alternative for tumor targeting. In gelatinase-expressing tumors, such as OV-90, targeted liposomal constructs, ^125^I-CTT2-SL and doxorubicin-containing CTT2, were found to be promising nanotherapeutic delivery vehicles for achieving therapeutic efficacy.  Table 1 summarizes the tumor uptakes of various targeted and nontargeted liposomal formulations. Differences in tumor uptake were observed range ovarian cancer models, with the largest uptake values (i.e., *∼*17% ID/g at 6 hrs) achieved in OV-90 hindlimb xenografts using CTT2-peptide-bound liposomes (*∼*500 peptides per liposome). Further, CTT2-bound micelles and liposomes, as well as the CTT2 peptide, demonstrated equivalent overall tumor uptake values, suggesting similar bioactivity. However, to achieve controlled and sustained drug release, we chose a nanoformulation instead of a prodrug approach (i.e., drug-peptide coupling). Our findings show that the utilization of these targeted nanoformulations results in a more efficient method for delivering therapeutics than passive (i.e., nontargeted) liposomal products (i.e., Caelyx). The development of CTT2-peptide-bound liposomes as a clinically promising targeting therapeutic that has the potential to improve drug delivery to human ovarian cancers will rest on the additional assessment of shelf and *in vivo* stability studies and formal toxicity testing.

## Figures and Tables

**Figure 3:**
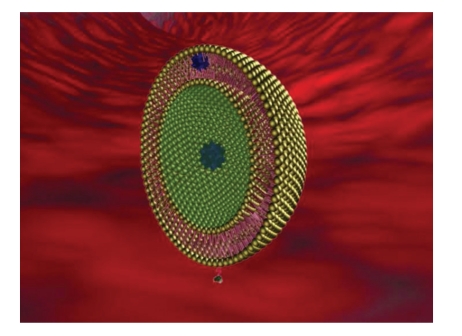
Schematic illustration of CTT2-PEG-3400-DSPE liposome [22].

**Table 1:** Tumor uptake of various liposomal constructs.

	Caelyx	CTT2-SL liposome
Targeted formulation	No	Yes
Concentration-targeted formulation	—	0.2%
Analyte	Doxorubicin	Doxorubicin
Time point (hours)	6	6
Tumor uptake (% ID/gram)	8.1%	19.0%

**Table 2:** 

			Lipid percent (%)
CTT2-SL liposome			
DSPE-mPEG2000	3.2 mg/ml	1.2 mM	5.5%
HSPC	9.6 mg/ml	12.2 mM	56.2%
Cholesterol	3.2 mg/ml	8.2 mM	38.1%
CTT2-PEG-lipid	0.2 mg/ml	0.04 mM	0.2%

Total lipids	16.2 mg/ml	21.6 mM	100.00%

Caelyx			
DSPE-mPEG2000	3.2 mg/ml	1.2 mM	5.5%
HSPC	9.6 mg/ml	12.2 mM	56.3%
Cholesterol	3.2 mg/ml	8.2 mM	38.2%

Total lipids	16.0 mg/ml	21.6 mM	100.0%

## References

[1] Torchilin V, Weissig  V (2003). *Liposomes: A Practical Approach*.

[2] Bendas G (2001). Immunoliposomes: a promising approach to targeting cancer therapy. *BioDrugs*.

[3] Nagaset H, Woessner JF (1999). Matrix metalloproteinases. Minireview. *The Journal of Biological Chemistry*.

[4] Curran S, Murray GI (2000). Matrix metalloproteinasesmolecular aspects of their roles in tumour invasion and metastasis. *European Journal of Cancer*.

[5] McCawley LJ, Matrisian LM (2001). Matrix metalloproteinases: they’re not just for matrix anymore!. *Current Opinion in Cell Biology*.

[6] Schmalfeldt B, Prechtel D, Härting K (2001). Increased expression of matrix metalloproteinases (MMP)-2, MMP-9, and the urokinase-type plasminogen activator is associated with progression from benign to advanced ovarian cancer. *Clinical Cancer Research*.

[7] Wu X, Li H, Kang L, Li L, Wang W, Shan B (2002). Activated matrix metalloproteinase-2 - A potential marker of prognosis for epithelial ovarian cancer. *Gynecologic Oncology*.

[8] Ozalp S, Tanir HM, Yalcin OT, Kabukcuoglu S, Oner U, Uray M (2003). Prognostic value of matrix metalloproteinase-9 (gelatinase-B) expression in epithelial ovarian tumors. *European Journal of Gynaecological Oncology*.

[9] Torng PL, Mao TL, Chan WY, Huang SC, Lin CT (2004). Prognostic significance of stromal metalloproteinase-2 in ovarian adenocarcinoma and its relation to carcinoma progression. *Gynecologic Oncology*.

[10] Lopata A, Agresta F, Quinn MA, Smith C, Ostor AG, Salamonsen LA (2003). Detection of endometrial cancer by determination of matrix metalloproteinases in the uterine cavity. *Gynecologic Oncology*.

[11] Davidson B, Goldberg I, Kopolovic J (1999). MMP-2 and TIMP-2 expression correlates with poor prognosis in cervical carcinoma—a clinicopathologic study using immunohistochemistry and mRNA in situ hybridization. *Gynecologic Oncology*.

[12] Talvensaari-Mattila A, Pääkkö P, Turpeenniemi-Hujanen T (2003). Matrix metalloproteinase-2 (MMP-2) is associated with survival in breast carcinoma. *British Journal of Cancer*.

[13] Ranuncolo SM, Armanasco E, Cresta C, De Kier Joffe EB, Puricelli L (2003). Plasma MMP-9 (92 kDa-MMP) activity is useful in the follow-up and in the assessment of prognosis in breast cancer patients. *International Journal of Cancer*.

[14] Toth M, Gervasi DC, Fridman R (1997). Phorbol ester-induced cell surface association of matrix metalloproteinase-9 in human MCF10A breast epithelial cells. *Cancer Research*.

[15] Brooks PC, Silletti S, von Schalscha TL, Friedlander M, Cheresh DA (1998). Disruption of angiogenesis by PEX, a noncatalytic metalloproteinase fragment with integrin binding activity. *Cell*.

[16] Jadhav U, Chigurupati S, Lakka SS, Mohanam S (2004). Inhibition of matrix metalloproteinase-9 reduces in vitro invasion and angiogenesis in human microvascular endothelial cells. *International Journal of Oncology*.

[17] Nguyen M, Arkell J, Jackson CJ (2001). Human endothelial gelatinases and angiogenesis. *International Journal of Biochemistry and Cell Biology*.

[18] Komatsu K, Nakanishi Y, Nemoto N, Hori T, Sawada T, Kobayashi M (2004). Expression and quantitative analysis of matrix metalloproteinase-2 and -9 in human gliomas. *Brain Tumor Pathology*.

[19] Koivunen E, Arap W, Valtanen H (1999). Cancer therapy with a novel tumor-targeting gelatinase inhibitor selected by phage peptide display. *Nature Biotechnology*.

[20] Penate Medina O, Söderlund T, Laakkonen LJ, Tuominen EKJ, Koivunen E, Kinnunen PKJ (2001). Binding of novel peptide inhibitors of type IV collagenases to phospholipid membranes and use in liposome targeting to tumor cells in vitro. *Cancer Research*.

[21] Penate Medina O, Kairemo K, Valtanen H (2005). Radionuclide imaging of tumor xenografts in mice using a gelatinase-targeting peptide. *Anticancer Research*.

[22] Medina OP, Zhu Y, Kairamo K (2004). Targeted liposomal drug delivery in cancer. *Current Pharmaceutical Design*.

[23] Kanda S, Inoue K, Nojima S (1982). Incorporation of ganglioside and spin-labelled ganglioside analogue into cell and liposome membranes. *The Journal of Biochemistry*.

[24] Uster PS, Allen TM, Daniel BE, Mendez CJ, Newman MS, Zhu GZ (1996). Insertion of poly(ethylene glycol) derivatized phospholipid into pre-formed liposomes results in prolonged in vivo circulation time. *FEBS Letters*.

[25] Ishida T, Iden DL, Allen TM (1999). A combinatorial approach to producing sterically stabilized (Stealth) immunoliposomal drugs. *FEBS Letters*.

[26] Zalipsky S, Mullah N, Harding JA, Gittelman J, Guo L, DeFrees SA (1997). Poly(ethylene glycol)-grafted liposomes with oligopeptide or oligosaccharide ligands appended to the termini of the polymer chains. *Bioconjugate Chemistry*.

[27] Dagar S, Sekosan M, Lee BS, Rubinstein I, Önyüksel H (2001). VIP receptors as molecular targets of breast cancer: implications for targeted imaging and drug delivery. *Journal of Controlled Release*.

